# Sequential Deposition of Integrated Cathode–Inorganic Separator–Anode Multilayers for High Performance Li-Ion Batteries

**DOI:** 10.1021/acsami.2c03828

**Published:** 2022-07-22

**Authors:** Jack D. Evans, Yige Sun, Patrick S. Grant

**Affiliations:** † Department of Materials, University of Oxford, Parks Road, Oxford OX1 3PU, U.K.; ‡ The Faraday Institution, Quad One, Becquerel Avenue, Harwell Campus, Didcot OX11 0RA, U.K.

**Keywords:** lithium-ion battery, spray processing, inorganic separator, direct deposition, layer-by-layer full cell

## Abstract

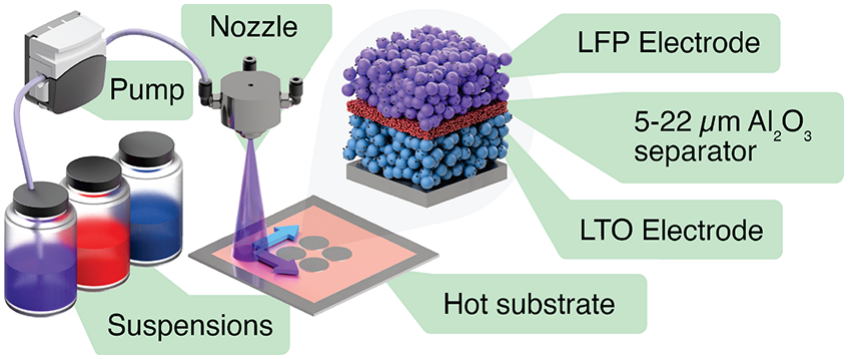

A porous, spray-deposited Al_2_O_3_-based separator was developed to enable the direct deposition of an electrode/separator/electrode Li-ion battery full cell assembly in a single operation. The optimized sprayed separator consisted of 50 nm Al_2_O_3_ particles, 1 wt % poly(acrylic acid), and 5 wt % styrene–butadiene rubber, deposited from an 80:20 vol % suspension of water and isopropanol. Separators between 5 and 22 μm thick had consistent and similar porosity of ∼58%, excellent wettability, thermal stability to at least 180 °C, adequate electrochemical stability and high effective ionic conductivity of ∼1 mS cm^–1^ at room temperature in an EC/DMC electrolyte, roughly double that of a conventional polypropylene separator. A sequentially deposited three-layer LiFePO_4_/Al_2_O_3_/Li_4_Ti_5_O_12_ full cell, the first of its kind, showed similar rate performance to an identical cell with a conventional polypropylene separator, with a capacity of ∼50 mAh g^–1^ at 30 C. However, after cycling at 2 C for 400 cycles, Al_2_O_3_ separator full cells retained 96.3% capacity, significantly more than conventional full cells with a capacity of 79.2% remaining.

## 1 Introduction

A Li-ion battery (LIB) is composed of current collectors (usually Al and Cu), electrodes (anode and cathode, consisting of active material, conductive additive, and binder), an electrolyte (a lithium salt in an organic solvent mixture), and a porous separator. The separator is a membrane designed to stop electronic contact between the anode and cathode while providing pathways for lithium ion transport, and this is conventionally achieved by using an porous electronic insulator, in which the pores become filled with the Li-ion conducting electrolyte.^1^ Therefore, it is favorable for these separators to have low electronic conductivity (to avoid anode–cathode short circuits), high affinity to electrolyte solvents, and high thermal, mechanical, and chemical stability and have a thickness that seeks to minimize lithium ion transport distance (typically 12–40 μm for commercial use).^1,2^

Most commercial LIB separators consist of microporous semicrystalline polyolefins, commonly polyethylene (PE), polypropylene (PP), or combinations of both (i.e., bilayer and trilayer).^3−5^ Commercial PE and PP separators are manufactured by using an extrusion process, either dry (e.g., Celgard) or wet.^3,6^ The dry process relies on forming pores between semicrystalline lamellar and amorphous regions in a polymer film by stretching, while the wet process relies on stretching before or after the removal of additives (which form pores on their removal).^3,6^ For the dry process, the shape of the pores is characteristic to the technique, that is, slit-shaped pores, while the distribution and size of pores can be controlled by processing conditions.^3^ Polyolefin separators have been favored due to low cost, a mature manufacturing process, good electrochemical stability, acceptable mechanical strength, and an intrinsic “thermal shutdown” capability.^7^ However, with the desire for higher performance and safety in LIBs, improvements to their relatively poor thermal stability (shrinkage at 90 °C) and still often poor wettability are a pressing requirement.^8,9^

Researchers have focused on two methods to produce separators with higher performance: (a) modification of existing polyolefins and (b) identification and application of alternative materials and manufacturing techniques.^4^ Surface coating, grafting, and blending of conventional polyolefin separators to improve wetting, thermal, mechanical, and chemical characteristics have been effective for many polymers,^10−13^ ceramics/inorganics,^14−17^ and combinations thereof.^18−21^ However, the improvements imparted by these modifications are often coupled with a complicated manufacturing process, increased separator thickness, a decrease in porosity, and/or debonding of modifying materials during cell assembly and cycling.^1,22,23^ Lee et al. provide an overview of the various modification techniques.^1^

Because of the issues and compromises associated with polyolefin modification, there have been efforts to identify alternative polymeric separators. High performance polymers, such as polyimide (PI),^24,25^ poly(ether ether ketone) (PEEK),^26^ polytetrafluoroethylene (PTFE),^27^ and poly(phenylene sulfide) (PPS)^28^ offer higher mechanical strength, good chemical and electrochemical resistance, and superior thermal resistance. Liu et al. showed that an electrospun polyimide/poly(vinylidene fluoride-*co*-hexafluoropropylene) (PI/PVDF-HFP) core/sheath separator could provide higher tensile strength (53 MPa), improved dimensional stability at 150 °C, and improved electrochemical performance over a Celgard PP separator.^29^ However, manufacture of separators based on higher performance polymers can be challenging due to their poor solubility in common solvents. Liu et al. recently showed that the thermally induced phase separation (TIPS) technique could overcome this challenge by preparing a thin (∼30 μm) PPS separator with high porosity (∼70%), excellent mechanical strength (∼120 MPa tensile strength and ∼7.5 GPa Young’s modulus), high thermal stability (>200 °C), and improved electrochemical performance in LFP half-cells over conventional PE.^28^

Ceramic and inorganic separators have also received attention due to their high wettability, excellent thermal stability, and comparatively high stiffness (to resist deformation).^7,14,30^ Common drawbacks include, in particular, the difficulty in creating a mechanically stable, thin, free-standing membrane and the often comparatively high material density (increasing the inactive mass in the cell).^31,32^ Takemura et al. presented the first example of a free-standing ceramic-based separator,^33^ where Al_2_O_3_ particles of 0.01 and 0.3 μm were combined with PVDF in varying ratios. The thermal shrinkage of the resulting separator was negligible up to 150 °C and decreased with reducing particle size and a higher powder-to-binder ratio. Cells based on an optimized oxide separator had similar capacity retention to cells using Celgard separators over 500 cycles.^33^ Zhang et al. produced a free-standing 37 μm Al_2_O_3_ separator from 100 to 300 nm particles, 6 wt % styrene–butadiene rubber (SBR), and 1 wt % poly(ethylene glycol) (PEG)^34^ and an aqueous process. On its own, SBR binder was unable to stabilize a free-standing film, while a combination of 1 wt % PEG and 6 wt % SBR produced a mechanically stable membrane. A subsequent heat treatment at 55 °C to remove the PEG resulted in dimensional stability at 130 °C for 40 min, an ∼0° contact angle with the electrolyte, and improved rate performance in LiNi_1/3_Co_1/3_Mn_1/3_O_2_/graphite full cells when compared with conventional PE.^34^

Most ceramic/inorganic particle separators are formed by using a slurry deposition method, so direct deposition onto the electrode is an obvious route to remove processing steps in the manufacture of LIBs. For example, Lagadec et al. stated, “With time, we expect separators to be viewed as a continuation of the electrodes themselves (perhaps even directly coated on them) with both structural and chemical functionality in mind.”^35^ Friesen et al. described how commercial LIB cells can make use of a 2–10 μm thick coating of 500 nm Al_2_O_3_ particles directly deposited onto LIB anodes in combination with a polymer separator, as an alternative to coating the polymer separator directly.^36^ Jung et al. directly coated 1–5 μm Li_7_La_3_Zr_2_O_12_ (LLZO) particles with 10–20 wt % PVDF-HFP directly onto graphite-based electrodes.^37^ A free-standing membrane of the same type had a thickness of 30 μm, a porosity of ∼40%, and no dimensional change after 1 h at 150 °C. Although the separator displayed a relatively poor electrolyte uptake of ∼20%, the rate and cycle performance were improved over cells using PP separators. Significantly, the LLZO separator suppressed thermal runaway in a pouch cell during nail penetration tests, while the PP separator cell surged to ∼280 °C. The thermal and dimensional stability of ceramic particles stopped direct contact between the anode and cathode, suggesting that in some cases and in comparison to conventional separators ceramic particle separators may improve cell safety.^37^ Other examples of direct deposition have shown similar improvements to thermal performance but generally produced membranes between 40 and 200 μm thick, which increases the inactive cell volume and mass considerably.^38−40^

Taking direct deposition further, Singh et al. showed that a layer-by-layer spray deposition technique enabled the fabrication of a functioning full cell including the current collectors, anode, cathode, and the separator.^41^ The separator consisted of PVDF, poly(methyl methacrylate) (PMMA), and fumed SiO_2_ at a ratio by weight of 27:9:4, deposited from a mixture of acetone and *N*,*N*-dimethylformamide (DMF), while the rest of the components were deposited from *N*-methyl-2-pyrrolidone (NMP). A separator thickness of 200 μm was said to be necessary to prevent internal shorting due to solvent penetration during deposition of the second electrode onto the separator. Despite the relatively thick separator, the cells showed good performance in an Li_4_Ti_5_O_12_–LiCoO_2_ (LTO–LCO) full cell for 65 cycles and could power LEDs after being printed onto the side of a mug.^41^

**Figure 1:**
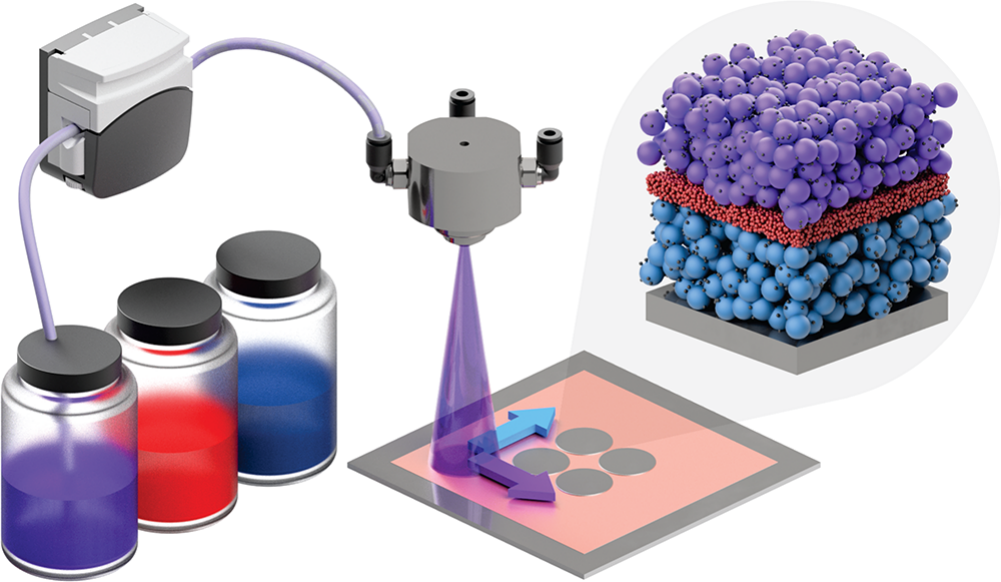
Schematic of the spray-printing process and the resulting microstructure of the sequential deposition of three different particle suspensions onto a stainless steel spacer to form a LIB cell.

The work described here advances previous examples of direct deposition of separators by providing a simple, potentially “greener” spray deposition route, paired with spray-deposited cathodes and anodes, for the sequential layer-by-layer processing of full cell assemblies. While inorganic layers have been used in combination with polymeric separators to improve thermal runaway and safety characteristics, an inorganic separator formed as an integral part of the manufacture of the full cell assembly has not been reported previously. The principal objective was to assess the promise of thin (5–22 μm) spray-deposited oxide layers in a simple electrode–separator–electrode integrated manufacturing sequence, as shown in Figure 1, which could enable the continuous sequential spray processing of LIBs. The spray-deposited separators were optimized in terms of dispersant/binder and suspension solvent, and room for further optimization remains. The proof-of-concept separators were tested with LiFePO_4_ (LFP) positive electrodes to assess their ability to enable stable cycling of positive electrode-based half-cells. The separator that yielded the best performance was then characterized further and compared with the performance of a commercial Celgard separator in LTO-based negative electrode half-cells for oxide separators of various areal densities. Spray-deposited full cells with a thin oxide separator layer were deposited via a continuous sequential procedure, which at the time of writing is the first report of this type, as similar prior work has instead relied on direct deposition of much thicker separators.^41^ We show that this arrangement provides improved wetting, thermal, rate, and degradation characteristics compared with a conventional Celgard 2500 polypropylene separator. Full experimental details are given in section 2.

## 2 Materials and Methods

### 2.1 Electrode Suspension Preparation

LiFePO_4_ (∼100 nm primary particle size, Hydro-Quebec, Canada) was used as cathode active material and Li_4_Ti_5_O_12_ (LTO; <200 nm, Sigma-Aldrich, UK) as the anode active material. Carbon black (C-ENERGY Super C65, Timcal Ltd., Switzerland) was used as a conductive additive, and sodium carboxymethylcellulose salt (CMC; *M*_w_ = 250000, degree of substitution 1.2, Sigma-Aldrich, UK) was used as the dispersant and binder. LFP and LTO suspensions had component ratios of 85:5:10 and 90:5:5 of active material:CMC:carbon black by mass, respectively. LFP and LTO slurries were mixed according to the following method: (1) carbon black and CMC solution were mixed by ball-milling for 15 min at 350 rpm by using 10 mm diameter ZrO_2_ balls, (2) active material and water were added until a slurry concentration of 40 wt % solid was reached and then mixed for a further 15 min at 350 rpm, (3) a portion of the slurry was extracted and diluted with water to a solid concentration of 1 g/dL, (4) the suspension was agitated via magnetic stirrer, and (5) 1 min before spray deposition, the solution was diluted with IPA to a solid concentration of 0.8 g/dL and solvent ratio of 80:20 vol % water:IPA.

### 2.2 Separator Suspension Preparation

Spray-deposited separators consisted of <50 nm Al_2_O_3_ (Sigma-Aldrich, UK), poly(acrylic acid) (PAA; *M*_v_ = 1250000, Sigma-Aldrich, UK), and styrene–butadiene rubber (SBR; MTI Corp. USA) as the oxide particles, dispersant, and binder, respectively. Suspensions with a oxide:dispersant:binder ratio of 94:1:5 were prepared as follows: (1) Al_2_O_3_, predissolved dispersant, and deionized water were added into a ball mill and milled for 10 min at 350 rpm with 10 mm diameter ZrO_2_ balls; (2) SBR solution was added, and the mixture ball-milled for a further 10 min at 350 rpm; (3) a slurry equal to 0.4 g of the solid component was diluted with water to a total volume of 80 mL and mixed for 1 min via magnetic stirring; (4) the diluted solution was sonicated for 5 min; and (5) the suspension was magnetically stirred and diluted with 20 mL of IPA to a final volume of 100 mL and a solvent ratio of 80:20 vol % water:IPA, 1 min before the spray deposition process.

### 2.3 Separator Suspension Characterization

Particle size and zeta-potential measurements of the suspended oxide particles were characterized by using dynamic and electrophoretic light scattering (DLS; Zetasizer, Malvern Instruments). For zeta-potential measurements, suspensions were prepared as above until step 4, at which point they were diluted with an aqueous solution of NaCl to reach an oxide particle concentration of 0.1 g/dL and 10 mM NaCl. For size measurement, the suspensions were diluted with deionized water only to 0.1 g/dL. Diluted suspensions were stirred by magnetic stirrer for 30 min before measurement.

### 2.4 Electrode and Separator Deposition

Electrode and separator layers were fabricated by using a spray-printing technique developed in our group, which has been described in detail previously^42−47^ and shown schematically in Figure 1. Briefly, an 80 mm × 80 mm Cu foil was attached to a heated vacuum chuck by polyimide tape, and ∼0.5 mm thick stainless steel spacers were placed on top. The temperature was set to 140 °C for deposition of electrodes or 150 °C for separator layers. The suspensions were fed through tubing by a Venturi pump at ∼3 mL min^–1^ and atomized in an industrial spray nozzle using compressed air at 0.3 bar. During atomization, the nozzle followed a preprogrammed zigzag (snake raster) pattern in the *x*–*y* plane over the spacers at a constant distance of 15 cm. As suspension droplets deposited on the hot spacers, the water and IPA components evaporated almost instantly to avoid resuspension of previously deposited material. Electrode material was deposited until an areal density of 2 and 2.3 mg cm^–2^ was achieved for LTO and LFP, respectively. All the data reported here are for uncalendered electrodes and layers, and the potential benefits or otherwise of calendering at various stages are discussed later.

For separator characterization, the separator was deposited from suspension directly onto spacers and Al foil. For half-cells, the separator was spray-deposited onto a preformed LTO electrode layer from suspension to coat the anode with a thickness of 5–22 μm. Full cells were formed by using the same process as that for half-cells up until the separator was deposited. Following this step, 3D-printed polymer masks were placed around the electrode–separator-coated spacers (to prevent connection of the second electrode with the cell casing around the cell perimeter), and the counter (LFP-based) electrode was then deposited. Full cell anode and cathode capacity was ensured to be a 1:1 ratio by measuring the spacers after deposition of each electrode and separator layer.

### 2.5 Electrode and Separator Physical Characterization

The mass and thickness of spacers and electrode/separator layers were measured by using an analytical balance with a readability of 0.1 mg (Sartorius Entris, Germany) and a micrometer with an accuracy of ±1 μm and a resolution of 1 μm (Mitutoyo 293 Series, Japan). Electrode surface morphology was characterized by scanning electrode microscopy (SEM; JEOL 6500F, Japan). Cross sections were prepared by using a plasma focused ion beam system (PFIB; FEI HELIOS, USA), and cross-sectional analysis was performed by using the built-in SEM and energy-dispersive X-ray spectroscopy (EDX) detectors and associated software.

The porosity was characterized by using nitrogen gas surface adsorption. Oxide separators deposited onto foil of ∼25 cm^2^ were evacuated and held under vacuum for 10 h in a surface area analyzer (Micromeritics Gemini V, USA). The sample was then immersed in liquid nitrogen and dosed aliquots of nitrogen for 37 steps of increasing pressure and then 30 steps of decreasing pressure. After each aliquot, pressure was equalized and the measurement performed. Manufacturer supplied software was used to model the resulting data to provide an estimation of surface area and pore width distribution.

Thermal properties of separators and their constituent components were characterized by thermogravimetric analysis (TGA; PerkinElmer Pyris, USA) in nitrogen gas from 25 to 900 °C at a 10 °C min^–1^ heating rate. For each sample, the TGA dish was coated with a bed of inert oxide powder to protect the Pt crucible from contamination. The Celgard 2500 separator was cut to the size of the dish, spray-deposited separators were scraped off the substrate, and powder samples were tested as-supplied. The mass loading of samples, dependent on the sample volume, was an average of ∼4 mg.

### 2.6 Cell Assembly

Cells were assembled according to the type of separator, the cell type, and the characterization techniques required, including separator-only cells, half-cells, and full cells for both conventional Celgard and spray-deposited separators. Separator-only cells (i.e., no anode or cathode) were assembled for linear sweep voltammetry and impedance testing. Unless stated otherwise, components were supplied by MTI Corp. USA.

Spray-deposited electrodes, Al_2_O_3_ separators, and combinations thereof were dried in a vacuum for 12 h at 150 °C in a vacuum oven (MTI Corp.) within a glovebox with an Ar atmosphere of <0.1 ppm of H_2_O and <0.1 ppm of O_2_. Half-cells, using Celgard 2500 separators as an example, were assembled with the following components placed one after another in the center of a stainless steel CR2032 coin cell cup: (1) a stainless steel spacer coated with working electrode (0.5 mm thick, 15.5 mm diameter), (2) 75 μL of LiPF_6_ EC/DMC = 50/50 (v/v) electrolyte (Sigma-Aldrich), (3) a Celgard 2500 separator (0.025 mm thick, 19 mm diameter), (4) 75 μL of LiPF_6_ EC/DMC = 50/50 (v/v) electrolyte, (5) Li foil (0.6 mm thick, 16 mm), (6) a stainless steel spacer (0.5 mm thick, 15.5 mm diameter), (7) a stainless steel wave spring (0.3 mm height), and (8) a stainless steel CR2032 coin cell cap. For Celgard full cells, the Li foil, i.e., component 5, was replaced with a spacer coated with a spray-deposited counter electrode. For linear sweep voltammetry, a spacer replaced the spray-deposited electrode, i.e., component 1. For impedance testing, both electrodes, i.e., components 1 and 5, were replaced with spacers.

For spray-deposited separator half-cells, 150 μL of electrolyte was dispensed on top of an electrode–separator-coated spacer and assembled from step 5. For three-layer spray-deposited full cells, a spacer coated with a working electrode, separator layer, and counter electrode was placed in the coin cell cup, 150 μL of electrolyte was dispensed on top, and assembly continued from step 6. For linear sweep voltammetry, a spacer coated with a separator layer was placed in the coin cell cup, 150 μL of electrolyte was dispensed on top, and assembly continued from step 5. For impedance testing, a spacer coated with a separator layer was placed in the coin cell cup, 150 μL of electrolyte was dispensed into the cell, a spacer was placed on top, and assembly was continued from step 6.

The assembled cells were crimped by using a coin cell crimper (MTI MSK-160E Electric Crimper, USA) and cleaned with ethanol after removal from the glovebox. Cells were allowed to rest for at least 20 h before initial cycling was performed.

### 2.7 Electrochemical Characterization

Electrochemical characterization was performed by using electrochemical impedance spectroscopy (EIS; Gamry Instruments Reference 600, USA), linear sweep voltammetry (Gamry Instruments Reference 600, USA), and galvanostatic cycling (Arbin Instruments BT-G-25 and IBT21084LC, USA).

The separator-only cell EIS response was measured between 200 kHz and 1 Hz at open circuit voltage, 20 h after construction. Fitting of separator EIS data was performed according to an R-CPE equivalent circuit, where R is a resistor in series with a constant phase element (CPE). The effective resistance of the electrolyte through the separator was estimated from the intersection of a best fit of the Nyquist plot with the real impedance axis. The effective ionic conductivity of the separator was calculated from the effective resistance and physical measurements of the separator according to
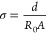
1where σ is the effective ionic conductivity, *d* is the separator thickness, *R*_0_ is the effective resistance of the electrolyte, and *A* is the separator area.

Linear sweep voltammetry measurements were collected on a potentiostat/galvanostat/ZRA system (Gamry Instruments Reference 600, USA). After resting for 20 h, spacer/separator/Li cells were subjected to a linear voltage sweep between 2 and 6 V vs Li/Li^+^ at a rate of 1 mV s^–1^.

LTO–Li coin cells were galvanostatically charged and discharged for three cycles at each rate between 0.1 and 1 C and cycled five times at each rate between 2 and 30 C before one cycle at 0.1 C. Long-term cycling was performed at 2 C for 400 cycles after rate cycling. Full cells of LTO–LFP were galvanostatically charged and discharged between 1 and 2.5 V, with two extra cycles at 0.1 C after rate cycling and seven cycles at 2 C to reach a steady state before long-term cycling. Capacity loss per cycle during long-term cycling was calculated by using linear regression for both half-cells and full cells.

## 3 Results and Discussion

### 3.1 Suspension, Substrate, and Deposition Optimization

To achieve a functional spray-deposited oxide separator, i.e., controllable and reproducible thickness, acceptable roughness, balanced density/porosity, and short resistant, an initial optimization was performed for the separator suspension, substrate choice, and deposition parameters.

#### Dispersant Selection

Table 1 shows the dispersion characteristics of the 50 nm Al_2_O_3_ particles used in the sprayed separators in water as a function of several common aqueous thickeners and pH modifiers. With no dispersant, the Al_2_O_3_ particles had a zeta-potential of 15.9 mV and an intensity-weighted mean hydrodynamic diameter (*Z*-average mean) of 421 nm at pH 7. On decreasing the pH to 4.3, the zeta-potential increased to 37.6 mV, suggesting greater suspension stability, and the *Z*-average mean reduced to 204 nm. Although altering the pH induced a more stable dispersion, acidic and alkaline conditions can corrode Al foil commonly used as one of the current collectors, and large pH modifications were not seen as viable for direct deposition of LIB separators. Carboxymethyl cellulose (CMC) is often favored in the literature as an aqueous binder due to its relatively low environmental impact. Adding 1 wt % CMC reduced the particle *Z*-average mean to 220 nm. However, a zeta-potential magnitude of only −19.8 mV suggested only limited stabilization. Alternatively, poly(acrylic acid) (PAA) improved the zeta-potential magnitude to −27.0 mV and provided an acceptable *Z*-average mean of 225 nm after 30 min. The magnitude of polydispersity in terms of additives was in the order none > pH > CMC > PAA, i.e., a narrowing of the particle diameter distribution and the degree of agglomeration from left to right. Therefore, on the basis of the similarity of the *Z*-average mean and the polydispersity index (PDI), but the improved (reduced) number mean diameter and (increased magnitude) zeta-potential over CMC, PAA was selected for further experimentation.

**Table 1:** Zeta-Potential and Particle Diameter Analysis for the 50 nm Al_2_O_3_ Particles Suspended in Water Using pH Modification, PAA (1 wt %), and CMC (1 wt %)

dispersant	zeta-potential (mV)	number mean^a^ (nm)	*Z*-average mean^b^ (nm)	PDI^c^
none	15.9 ± 4.9	159	421	0.49
pH 4.3	37.6 ± 6.5	74	204	0.39
PAA	–27.0 ± 8.4	93	225	0.35
CMC	–19.8 ± 7.3	110	220	0.36

a Number mean diameter.

b Intensity-weighted mean hydrodynamic diameter.

c Polydispersity index.

#### Binder Optimization

**Figure 2:**
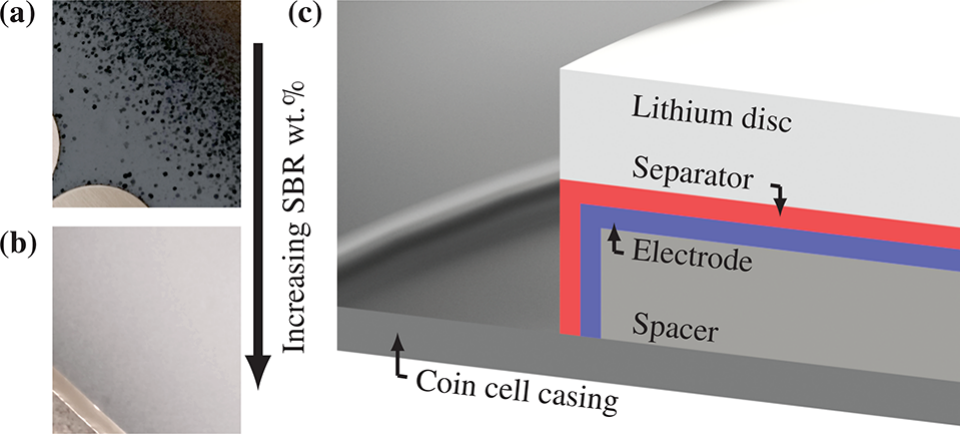
Optimization of binder and substrate for spray-deposited cells. Optical micrograph of Al_2_O_3_ nanoparticle coatings deposited onto LFP electrodes from suspensions containing (a) 2 wt % SBR and (b) 5 wt % SBR by solid mass. A spray-deposited separator half-cell with (c) the stainless steel spacer current collector creating a large separation between the lithium disc and the cell casing to avoid short circuits in test cells.

Styrene–butadiene rubber (SBR) was selected as a model binder due to its known compatibility and widespread use in aqueous manufacture of LIB anodes. SBR emulsion is insoluble in water upon drying, which reduced the risk of suspension of previously deposited layers in subsequent droplets. Suspension of previously deposited material could have caused short circuits by generating pathways of conducting material (i.e., carbon black) through the separator layer during deposition. Figure 2a shows that at 2 wt % SBR the sprayed Al_2_O_3_ tended to flake off in small circular areas, likely caused by tensile capillary forces during drying. Figure 2b shows that when the binder was increased to 5 wt % or greater, the Al_2_O_3_ particles maintained adhesion to the electrode layer and substrate.

#### Substrate Selection

Stainless steel circular spacers from 2032 coin cells were selected as substrates to facilitate simple, punch-less assembly into standard cells. In assembling coin half-cells with an electrode and separator deposited on thin Al foil, an Li chip of diameter smaller than the separator and with exact alignment must be used to avoid short circuits. Alternatively, the use of the spacer raised the contact point of the Li foil into the middle of the cell and retained the protective conformal coating of the separator layer, removing the chance of contact between the Li foil and the cell cup or working electrode, as shown in Figure 2c. In terms of full cells, it was assumed that punching spray-deposited full cells could induce a short circuit by smearing material across the sides of the separator, potentially connecting the anode and cathode, and was avoided.

#### Solvent Optimization

A separator with a uniform structure and low roughness was desired (i.e., with no “bumps” or cracks). Separators with excessively heterogeneous structures will result in a heterogeneous current density distribution, leading to localized heating and degradation of the electrode and separator, which could lead to physical contact and short circuits between the anode and cathode.^48,49^

**Figure 3:**
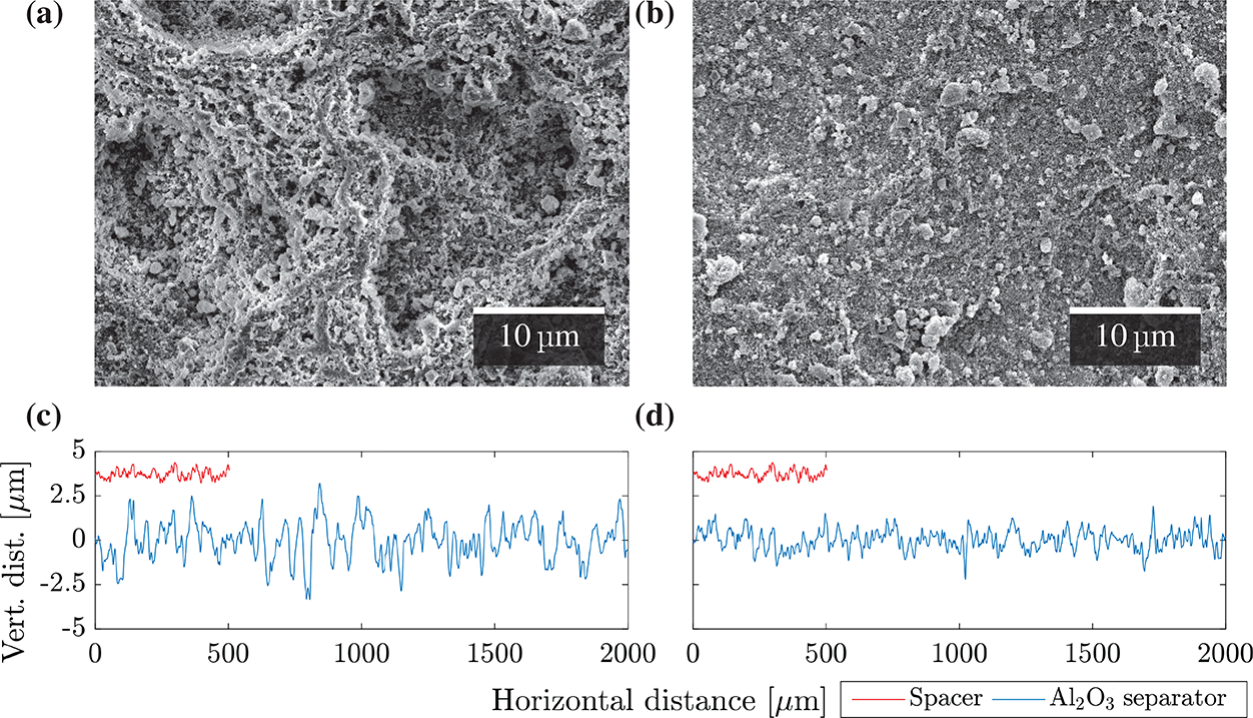
Plan-view SEM micrographs of Al_2_O_3_ layers deposited on top of sprayed LFP-based electrodes and corresponding surface profiles. Al_2_O_3_ was deposited from (a) water and (b) water:IPA 80:20 vol % suspensions containing 1 wt % PAA and 5 wt % SBR of the total solid mass. Surface profiles of (a) and (b) are displayed in (c) and (d), respectively.

Figure 3a shows the surface of an Al_2_O_3_ separator deposited from water alone (W) onto an LFP electrode, while Figure 3b shows the surface of Al_2_O_3_ deposited from water with 20 vol % IPA (IPA), which had a qualitatively smoother surface. Some agglomerates persisted, most with a diameter of 1–3 μm, up to a maximum of ∼4 μm.

Surface roughness measurements of Al_2_O_3_ (W) and Al_2_O_3_ (IPA) are shown in Figures 3c and 3d, respectively, and the roughness measurements are summarized in Table 2. Table 2, consistent with the micrographs, shows that water with 20 vol % IPA gave the lowest coating roughness. The Al_2_O_3_ separator deposited from water alone had a lower areal density and greater thickness, which were likely due to the increased roughness. Droplets of water commonly deposit suspended particles in heterogeneous patterns due to a weak Marangoni flow during evaporation.^50^ A mixture of low and high boiling point liquids can suppress the effect, which could explain the improvement gained by including 20 vol % IPA in the suspension. Overall, the physical properties of Al_2_O_3_ deposited from water with 20 vol % IPA were favored over that of Al_2_O_3_ deposited from water alone.

**Table 2:** Physical Properties of Oxide Separators, Al_2_O_3_ Deposited from Water Alone (W), and Al_2_O_3_ Deposited from Water with 20 vol % Isopropyl Alcohol (IPA)^a^

separator	*R*_a_ (μm)	*W*_a_ (μm)	max(P2V) (μm)	areal density (mg cm^–2^)	thickness (μm)	FC
Al_2_O_3_ (W)	0.5	1.0 ± 0.3	5.3 ± 0.8	1.59 ± 0.05	12 ± 1	0/3
Al_2_O_3_ (IPA)	0.3	0.7 ± 0.2	3.7 ± 0.7	1.70 ± 0.05	11 ± 1	3/3

a *R*_a_ is the arithmetic average deviation from the mean line, *W*_a_ is the arithmetic average deviation of waviness from the mean line, and max(P2V) is the largest height difference between a neighboring peak and valley. The functional cells (FC) column lists how many of three assembled cells were able to charge and discharge on the first cycle.

Three LFP half-cells with sprayed separators from each solvent mixture were assembled. The LFP half-cells with Al_2_O_3_ deposited from water alone could not charge due to short circuits between the anode and cathode caused by significant variations in separator thickness. In contrast, the preferred Al_2_O_3_ separator deposited from water with 20 vol % IPA mitigated any short circuits and was chosen for subsequent in-depth comparison with a commercial polymer separator.

### 3.2 Physicochemical Properties of Spray-Deposited Al_2_O_3_ Separators

Given their encouraging proof-of-concept demonstration when sprayed onto a cathode, spray-deposited Al_2_O_3_ separator physical properties, thermal stability, wettability, impedance, and electrochemical performance in LTO-based (anode) half-cells were investigated. A Celgard 2500 PP monolayer separator was used for comparative purposes.

#### Physical Characterization

**Figure 4:**
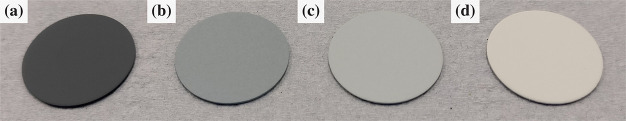
Stainless steel spacers coated with (a) an LTO electrode layer and (b–d) an LTO electrode layer coated with a 5, 11, and 22 μm thick Al_2_O_3_ separator layer, respectively.

Suspensions comprising of 50 nm Al_2_O_3_ particles, PAA, SBR in a 94:1:5 solid ratio, and 80:20 vol % water:IPA solvent were spray-deposited onto spacers precoated with an ∼14 μm LTO-based electrode. Figure 4 shows the resulting coated spacers, where (a) is the spacer/LTO electrode and (b–d) are spacer/LTO/Al_2_O_3_ electrode/separator composites, where the approximately 5, 11, and 22 μm thick Al_2_O_3_ separator layers are termed Al_2_O_3_ (5 μm), Al_2_O_3_ (11 μm), and Al_2_O_3_ (22 μm) where needed, respectively. Table 3 compares the areal density, thickness, and estimated porosity of the Al_2_O_3_ separators with Celgard 2500. The relatively high density of Al_2_O_3_ (3.95 g cm^–3^) resulted in a higher mass per unit volume (PP density assumed at 0.9 g cm^–3^). To obtain the same areal density, or better, than Celgard 2500, the Al_2_O_3_ separator should be ∼7 μm thick, i.e., approximately a third to a quarter of the Celgard thickness, assuming similar porosity. The Al_2_O_3_ separator porosity was estimated as 58 ± 3, 59 ± 5, and 60 ± 10% for 22, 11, and 5 μm thickness, respectively, slightly higher than that of Celgard 2500.^51,52^

**Table 3:** Comparison of the Thickness, Mass, Estimated Porosity, and Pore Size of a Celgard 2500 Separator and Three Spray-Deposited Al_2_O_3_-Based Separators of Varying Thickness^51,52^

separator	AD^a^ (mg cm^–2^)	*d*^b^ (μm)	porosity (%)	pore size (μm)
Celgard	1.13 ± 0.04	25 ± 1	55	0.21 × 0.06
Al_2_O_3_ (5 μm)	0.74 ± 0.05	5 ± 1	60 ± 10	
Al_2_O_3_ (11 μm)	1.70 ± 0.05	11 ± 1	59 ± 5	
Al_2_O_3_ (22 μm)	3.44 ± 0.05	22 ± 1	58 ± 3	

a Areal density.

b Thickness.

**Figure 5:**
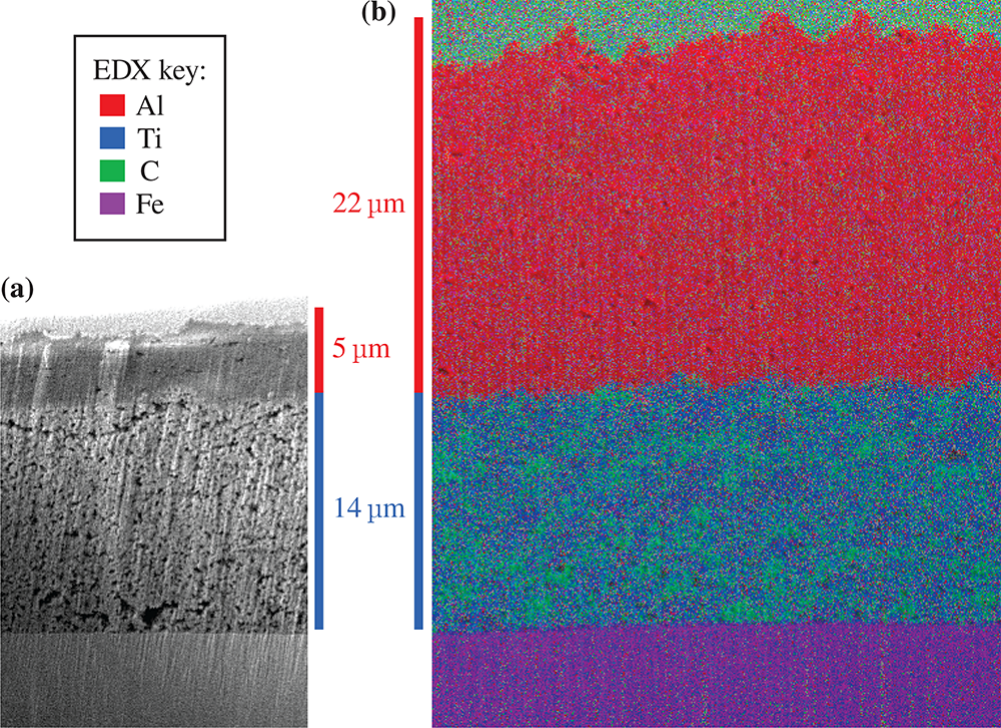
Cross-sectional SEM images of LTO-based electrodes coated with (a) Al_2_O_3_ (5 μm) and (b) Al_2_O_3_ (22 μm) with an overlaid EDX map. From bottom to top, the layers are the stainless steel spacer (purple), the LTO (blue) and carbon black (green) electrode, the Al_2_O_3_ separator (red), and the Pt/C coating layer.

Figures 5a and 5b show SEM cross-sectional images of ∼14 μm LTO electrodes coated with 5 and 22 μm thick Al_2_O_3_ separators. The Al_2_O_3_ separator coated conformally onto the underlying sprayed LTO-based layer. From Figure 5a,b, Al_2_O_3_ (5 μm) had an estimated deviation from the mean line of ±1 μm, while Al_2_O_3_ (22 μm) was slightly rougher at ∼±2 μm. Al_2_O_3_ (5 μm) had a few large pores of diameters of 200–300 nm, while Al_2_O_3_ (22 μm) had pores with diameters up to ∼400 nm.

Nitrogen gas surface adsorption (BET) was used to investigate the specific surface area and mesoporosity of Al_2_O_3_ separator layers on Al foil. A separator layer fabricated from 300 nm Al_2_O_3_ particles was manufactured to provide additional comparison. A separator consisting of 300 nm Al_2_O_3_ was unable to hold charge when tested in LFP half-cells, suggesting that particle size (or pore diameter) was likely a critical factor when successfully fabricating spray-deposited separators.

**Figure 6:**
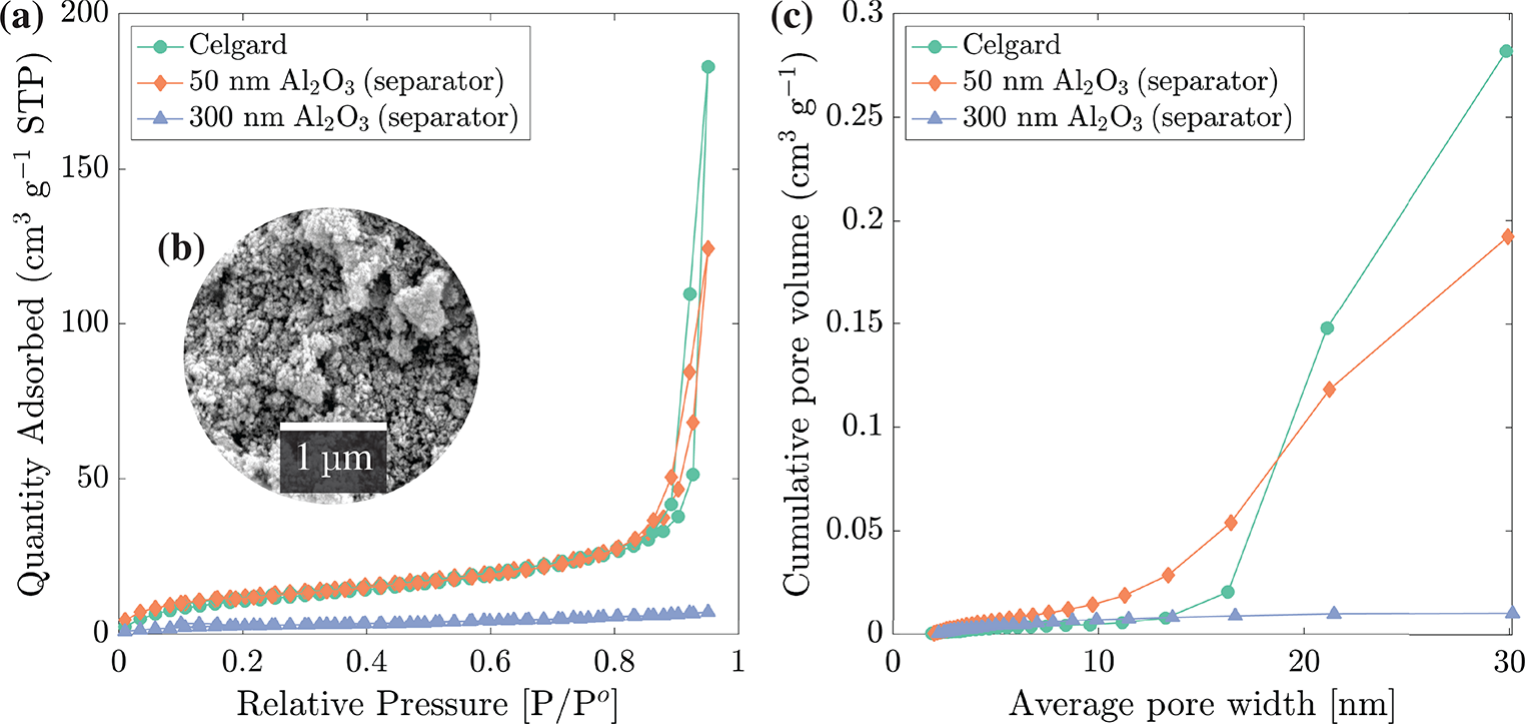
Nitrogen gas adsorption measurements and calculations for Celgard 2500 and separators fabricated by using 50 nm Al_2_O_3_ and 300 nm Al_2_O_3_. (a) BET isotherms, (b) micrograph of the separator using 50 nm Al_2_O_3_, and (c) BJH desorption cumulative average pore width distributions.

The BET isotherms of the Celgard and separators fabricated by using 50 nm Al_2_O_3_ and 300 nm Al_2_O_3_ are shown in Figure 6a. Celgard and the separator using 50 nm Al_2_O_3_ displayed a hybrid shape between a classical type II and type IVa behavior,^53^ comprising adsorption–desorption hysteresis.

According to IUPAC, type II behavior is typical of nonporous or macroporous solids, as multilayer formation of adsorbed nitrogen appears to increase without limit as *p*/*p*° tends to 1. A type IVa isotherm is typical of mesoporous solids and is generated due to pore condensation, where the adsorbing gas condenses to a liquidlike state at a pressure below *p*°. However, a final saturation plateau, typical of a type IVa, was not observed, indicating a structure with both mesoporosity and macroporosity.^53^

H1 hysteresis is usually associated with a narrow range of uniform pores. However, it has also been attributed to spherical/cylindrical pore structures with wide openings through which there is immediate access to the vapor phase.^53,54^ The distinctive isotherm of the separator using 50 nm Al_2_O_3_ likely represents a porous powder bed with little or no pore blocking, similar to isotherms of nonporous nanoparticles.^55−57^

The specific surface area (SSA) was calculated from the BET isotherms and is shown in Table 4. For the separator using 300 nm Al_2_O_3_ SSA = 9.4 m^2^ g^–1^ with no distinctive hysteresis loop, indicating little or no mesoporosity. For the separator using 50 nm Al_2_O_3_ SSA = 44.4 m^2^ g^–1^, while for Celgard SSA = 41.3 m^2^ g^–1^. The separator using 50 nm Al_2_O_3_ had an estimated density of 3.77 g cm^–3^, ∼4 times greater than that of Celgard at 0.9 g cm^–3^, so the difference in the surface area per unit volume was much higher than Table 4 suggested. A high surface area could increase electrolyte uptake but may also increase chemical side reactions.

**Table 4:** Comparison of the Specific Surface Area of the Celgard Separator and the Separators Using 50 and 300 nm Al_2_O_3_ and the Calculated Proportion of Pore Widths in the Celgard and Separator Using 50 nm Al_2_O_3_^a^

separator	SSA (m^2^ g^–1^)	*V*_sep_	*V*_solid_	*V*_pore_	*V*_meso_	*V*_macro_	*V*_meso,%_	*V*_macro,%_
300 nm Al_2_O_3_	9.4							
Celgard	41.3	2.47	1.11	1.36	0.28	1.08	21	79
50 nm Al_2_O_3_	44.4	0.60	0.27	0.34	0.20	0.14	59	41

a On a per gram basis, *V*_sep_ is the volume of the separator, and *V*_solid_, *V*_pore_, *V*_meso_, and *V*_macro_ are the volumes occupied by the solid, total porosity, mesopore, and macropore components of the separator, respectively, with units of cm^3^ g^–1^. *V*_meso,%_ and *V*_macro,%_ are the percentage of total porosity credited to meso and macropores, respectively.

Figure 6c shows that the separator using 300 nm Al_2_O_3_ had little mesoporosity, represented by a low cumulative mesopore volume of 0.01 cm^3^ g^–1^. The cumulative mesopore volume of the Celgard and the separator using 50 nm Al_2_O_3_ were 0.28 and 0.20 cm^3^ g^–1^, respectively. The Celgard separator showed a narrower pore width distribution (PWD) with an average pore width of 22 nm. The separator using 50 nm Al_2_O_3_ had an average pore width of 17 nm and a broader PWD in the mesopore range, with minimum pore widths of <10 nm.

To estimate the volume of each component in the separators, a simple relation was used:
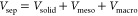
2where, on a per gram basis, *V*_sep_ is the volume of the separator, and *V*_solid_, *V*_meso_, and *V*_macro_ are the volumes occupied by the solid, mesopore, and macropore components of the separator, respectively, as shown for the Celgard and Al_2_O_3_ separators in Table 4. Table 4 reinforces some important comparisons between the separator using 50 nm Al_2_O_3_ and Celgard. Celgard had a significantly larger pore volume per gram of material (*V*_pore_) of 1.36 cm^3^ g^–1^ compared with that of Al_2_O_3_ at 0.34 cm^3^*g*^–1^, displaying an advantage of polymer-based separators. The ratio of mesopores to macropores was 0.59:0.41 for the separator using 50 nm Al_2_O_3_, compared with 0.21:0.79 for the Celgard separator. The proportion of mesopores in the separator using 50 nm Al_2_O_3_, assuming a monomodal pore distribution, suggested a fine-scale average pore width near that of the average particle diameter of ∼50 nm.

#### Temperature Stability

**Figure 7:**
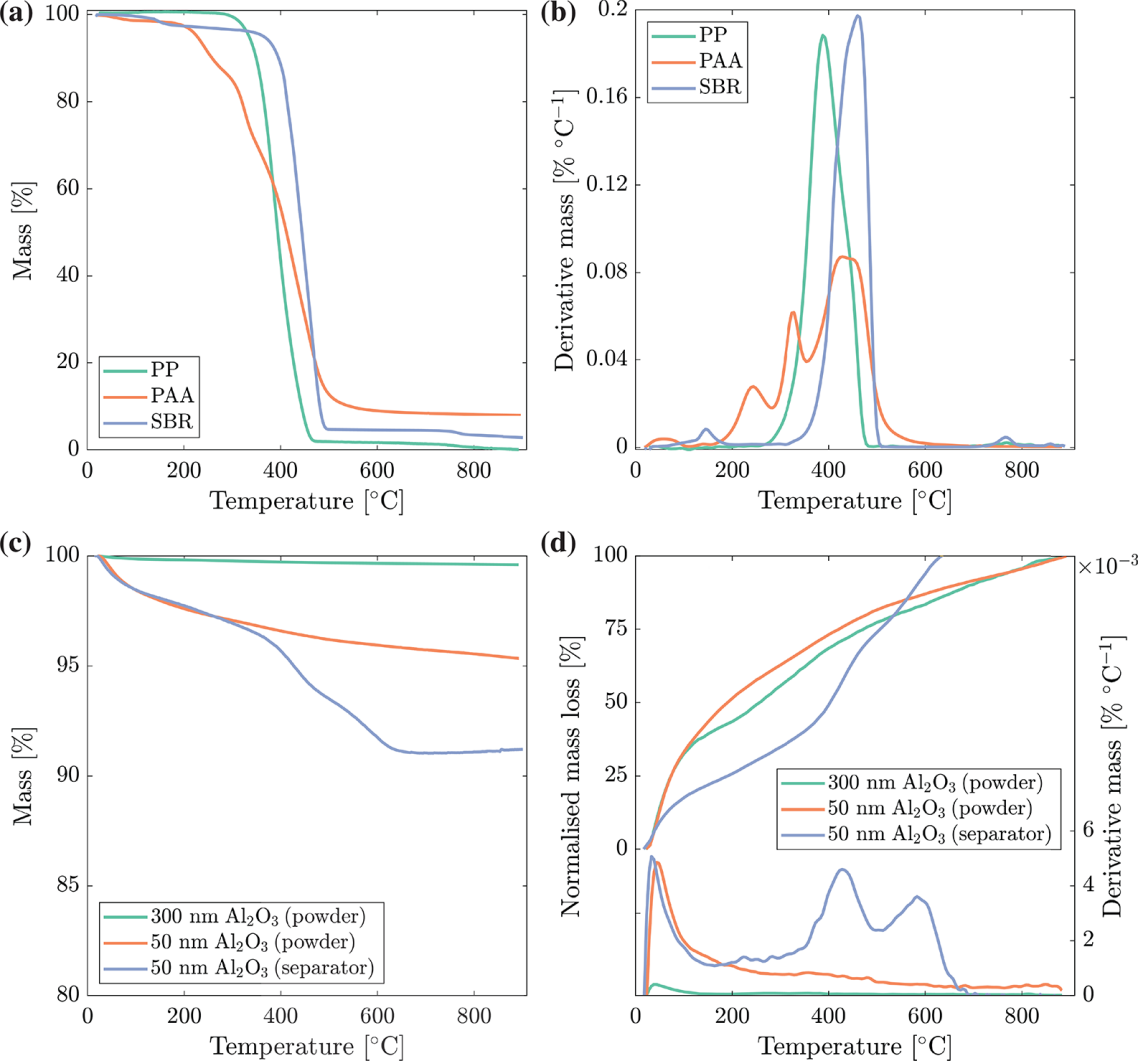
Thermogravimetric analysis of a Celgard 2500 separator and a spray-deposited Al_2_O_3_ separator and requisite materials between 25 and 900 °C. (a) TGA and (b) DTG of Celgard 2500 (PP), poly(acrylic acid) (PAA), and styrene–butadiene rubber (SBR). (c) TGA, (d, upper) normalized mass loss, and (d, lower) DTG of 300 nm Al_2_O_3_ powder, 50 nm Al_2_O_3_ powder, and spray-deposited Al_2_O_3_ separator using 50 nm Al_2_O_3_ particles.

Figure 7 shows TGA plots for the Celgard 2500 separator (Celgard), poly(acrylic acid) (PAA), styrene–butadiene rubber (SBR), 300 nm Al_2_O_3_ and 50 nm Al_2_O_3_ powders, and the spray-deposited separator using 50 nm Al_2_O_3_ between 25 and 900 °C. Figure 7a,b shows that PP decomposed in a single step at 252 °C, with a maximum rate of degradation (DTG peak) at 387 °C, and was fully degraded at 482 °C. Poly(acrylic acid) displayed four distinct DTG peaks, with three well-documented high-temperature peaks.^58−60^ The first peak (2% mass loss at 50–130 °C) was simply evaporation of absorbed water. Stage 1 of polymer decomposition (mass loss of 11% at 160–280 °C with a peak at 240 °C) was attributed to reversible anhydride formation.^61^ Stage 2 (mass loss of 22% with a peak at 330 °C) was due to decarboxylation of the anhydride and release of CO_2_. Finally, a broad peak, related to chain scission of the polymer, occurred above 350 °C, and led to a further mass loss of 55%. A mass of 10% carbonaceous residue remained above 530 °C. Styrene–butadiene rubber decomposed in a single step between 320 and 520 °C with a DTG peak at 460 °C and left a residual mass of 4.5%. The small peak at 150 °C was most likely due to volatilization of processing additives.^62^

The 300 nm Al_2_O_3_ powder in Figure 7c maintained 99.6% mass up to 900 °C. The 50 nm Al_2_O_3_ powder reduced in mass gradually to 95.3% at 900 °C. Figure 7d shows the normalized mass loss against temperature (upper traces, left axis) and the DTG (lower traces, right axis). Both 50 and 300 nm Al_2_O_3_ powders lost mass below 100 °C, which was attributed to evaporation of physically adsorbed water. In terms of normalized mass loss, both powders lost 35% of their total mass loss below 100 °C and followed similar trends up to 900 °C. An equilibrium between aluminum oxide–hydroxides and Al_2_O_3_ occurs at ∼370 °C (below 200 bar), and this may be evident in the DTG of 50 nm Al_2_O_3_ and the normalized mass loss of 300 nm Al_2_O_3_.^63^ Therefore, the slight differences between the Al_2_O_3_ powders above 150 °C were most likely due to slightly different hydroxide surface species, and overall the mass loss of 50 nm Al_2_O_3_ powder was as expected.

The optimized Al_2_O_3_ separator comprised a 94:1:5 composite of 50 nm Al_2_O_3_ particles, PAA and SBR, and displayed similar mass loss behavior to 50 nm Al_2_O_3_ powder until 300 °C, along with some underlying degradation of the small quantity of the PAA consistent with Figure 7a,b. There were two large decomposition peaks in Figure 7d, at 420 and 600 °C, the last of which in particular was not previously resolved in the individual constituent materials. Decomposition continued until a temperature (∼720 °C) higher than the polymers by themselves (620 °C), potentially due to the reduced thermal conductivity of the composite separator or the degradation of some new species formed between the Al_2_O_3_, the Al_2_O_3_ surface species, and/or one or more of the polymer components at lower temperature. At 900 °C, the 50 nm Al_2_O_3_ powder had 95.3% of original mass remaining, while the separator using 50 nm Al_2_O_3_ retained 91.2%. This difference was slightly less than expected from the 6 wt % of PAA and SBR combined in the oxide separator but lies within the likely error of the technique.

#### Electrolyte Wetting

**Figure 8:**
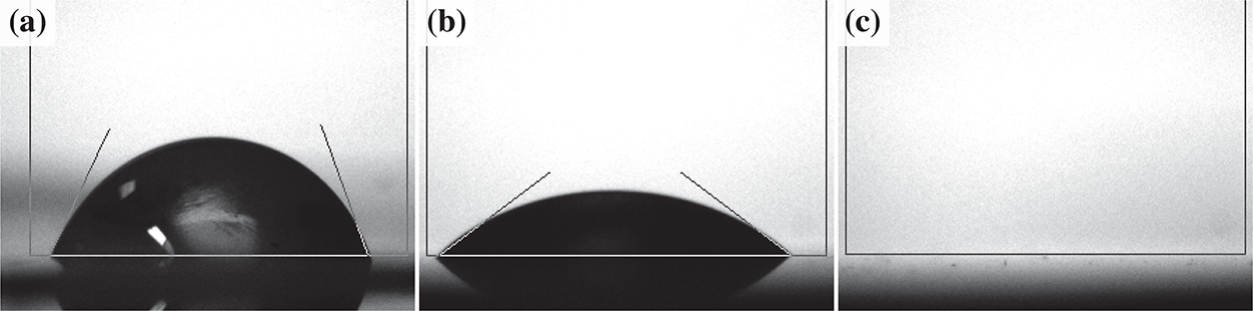
Contact angle measurements of ethylene carbonate (EC) and dimethyl carbonate (DMC) in a 50/50 v/v mixture dropped onto (a) Celgard 2500, (b) a stainless steel spacer, and (c) a stainless steel spacer coated with the spray-deposited Al_2_O_3_ separator.

The performance of separators relies significantly on their wettability with the electrolyte.^10,64,65^ A high contact angle (poor wetting) is usually expressed as a relatively high electrolyte resistance across the separator and unfilled pores may result. Figure 8 shows contact angle measurements of a 50/50 v/v mixture of ethylene (EC) and dimethyl carbonates (DMC) on the Celgard 2500 separator, a stainless steel spacer, and a stainless steel spacer coated with a separator using 50 nm Al_2_O_3_, with contact angles of 64.9 ± 5.5°, 35.8 ± 1.2°, and ∼0°, respectively. A coating of 50 nm Al_2_O_3_ only 5 μm thick reduced the contact angle of the spacer from 36° to ∼0°.

### 3.3 Electrochemical Properties of Spray-Deposited Al_2_O_3_-Based Separators

#### Separator Impedance

**Figure 9:**
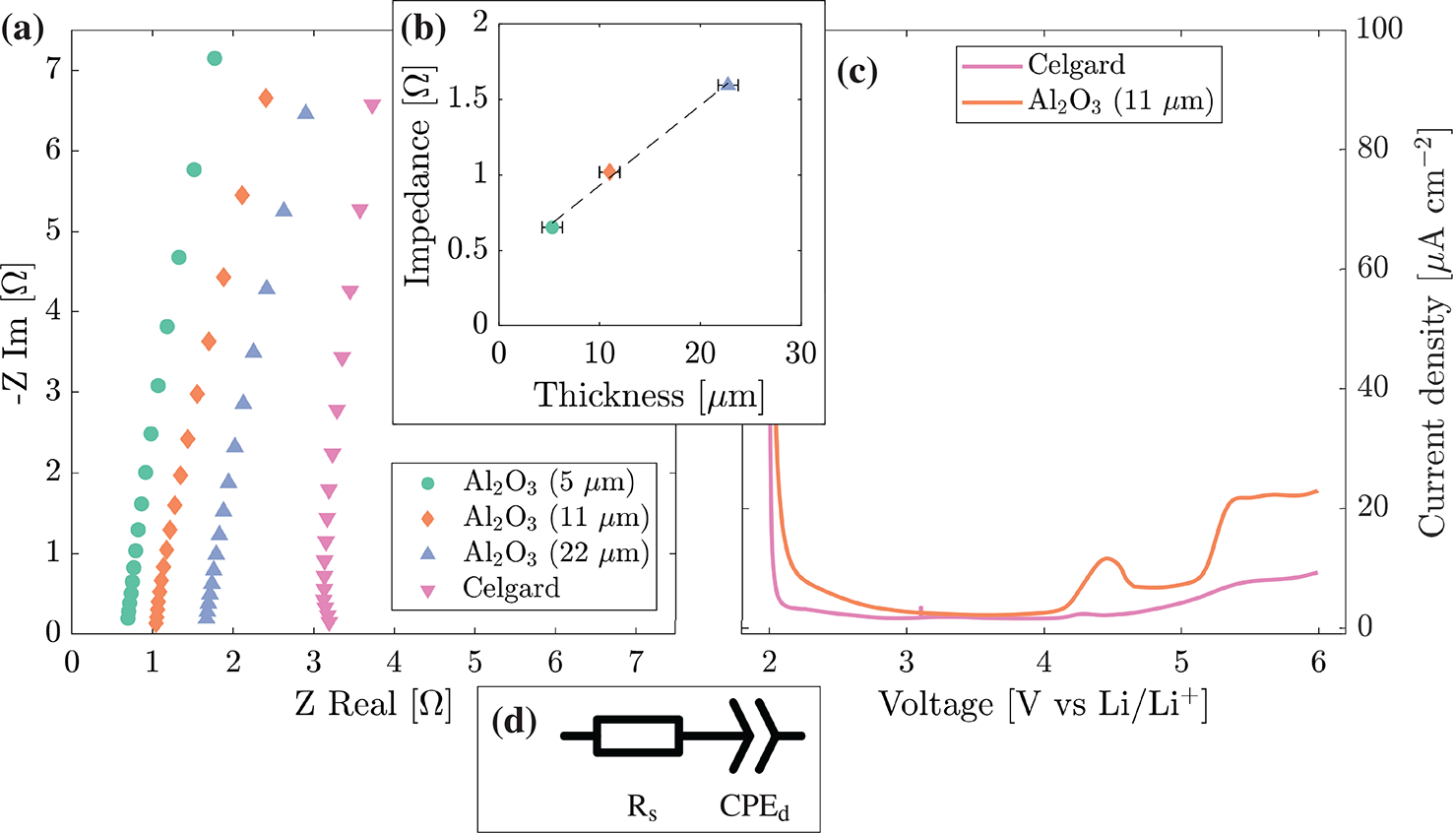
Impedance spectroscopy and linear voltage sweep response of separators soaked in a 1.0 M LiPF_6_ EC/DMC 50/50 v/v electrolyte. (a) Impedance spectroscopy between 10 and 200 kHz for Celgard and 5, 11, and 22 μm thick Al_2_O_3_ separators, (b) the relationship between equivalent series resistance and Al_2_O_3_ separator thickness, (c) linear voltage sweeps for Celgard and the 11 μm thick Al_2_O_3_ separator, and (d) the equivalent circuit used to estimate the equivalent series resistance from (a).

Figure 9a shows Nyquist plots from electrochemical impedance spectroscopy (EIS) to estimate the effective ionic conductivities of electrolyte soaked separators in spacer/separator/spacer coin cells. From this point, all Al_2_O_3_ separators were fabricated with 50 nm Al_2_O_3_. The equivalent series resistance, determined by modeling the impedance response by using Figure 9d, was 0.35, 0.54, 0.84 and 1.62 Ω cm^–2^ for 5, 11, and 22 μm thick Al_2_O_3_ separators and a Celgard separator. Figure 9b plots the equivalent series resistance against the separator thickness and shows an approximately linear relationship for the Al_2_O_3_ separators. The effective ionic conductivity of the Al_2_O_3_ separators was estimated as 0.98 ± 0.05 mS cm^–1^, roughly double that of Celgard 2500 of 0.50 mS cm^–1^. The higher ionic conductivity and lower equivalent series resistance were attributed to the superior wettability of the Al_2_O_3_ separator.

#### Electrochemical Stability

The stability of the separators under electrochemical conditions was studied by using linear sweep voltammetry on spacer/separator/spacer coin cells in 1 M LiPF_6_ EC/DMC 50/50 v/v electrolyte, as shown in Figure 9c. The Celgard separator showed a current density of ∼1.7 μA cm^–2^ up to 4 V and then a continual rise in current density after ∼4.5 V. The small peak in current density at 4.2–4.3 V was due to the oxidation of water (based on the standard hydrogen potential).^66,67^ The current density of the Al_2_O_3_ separator cell up to 4 V was ∼2.4 μA cm^–2^, slightly higher than that of the Celgard separator cell.

The water oxidation peak for the Al_2_O_3_ separator cell was relatively large at 11.6 μA cm^–2^ over 4.2–4.6 V and was likely due to the difficulty in removing excess water from the hydrophilic PAA dispersant and high surface area Al_2_O_3_ particles. The current density of the Al_2_O_3_ separator cells reduced to 7.5 μA cm^–2^ at ∼5 V, close to 4.5 μA cm^–2^ of the Celgard separator cells. Above 5.1 V, the current density for the Al_2_O_3_ separator was ∼2 times that of Celgard. Overall, the Al_2_O_3_ separator displayed adequate voltage stability up to 5 V, but there may be room for further improvement with the use of more stable polymer binders and improved drying procedures.

#### Galvanostatic Cycling

**Figure 10:**
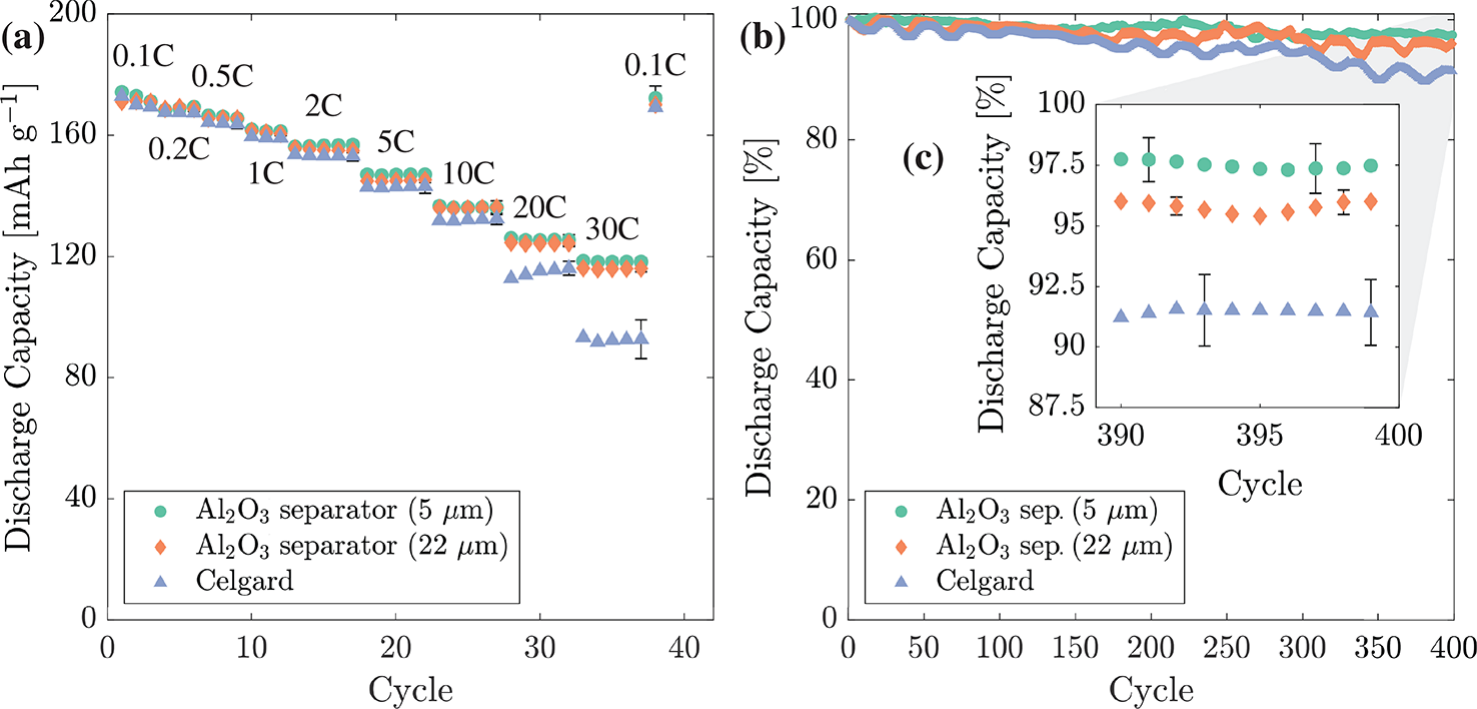
Comparison of LTO half-cell performance using Celgard or Al_2_O_3_ separators: (a) cycling performance between 0.1 and 30 C, (b) long-term cycling at 2 C for 400 cycles, and (c) a magnified inset of the last 10 cycles in (b).

The performance of LTO/separator/Li half-cells containing Al_2_O_3_ (5 μm), Al_2_O_3_ (22 μm), and Celgard were investigated between 0.1 and 30 C, as shown in Figure 10a. The 5 and 22 μm thick Al_2_O_3_ separators produced consistent capacities with a maximum difference in capacity of 2% at 30 C. Between 0.1 and 10 C, all types of separators facilitated an initial discharge capacity of 170 ± 2 mAh g^–1^, with the largest difference in capacity of only 2.8% between the Al_2_O_3_ separators (136.2 ± 1.0 mAh g^–1^) and the Celgard separator (132.5 ± 1.9 mAh g^–1^) at 10 C. However, at higher rates, Al_2_O_3_ (5 μm) outperformed Celgard by 8.1% with a capacity of 125.5 ± 1.0 mAh g^–1^ compared with 116.1 ± 2.3 mAh g^–1^ at 20 C and by 27.8% with a capacity of 118.3 ± 1.2 mAh g^–1^ compared with 92.5 ± 6.4 mAh g^–1^ at 30 C. Similarly, Al_2_O_3_ (22 μm) was within ±2 mAh g^–1^ of Al_2_O_3_ (5 μm) at all tested C rates. The Al_2_O_3_ separators evidenced a relatively low standard deviation between cells, which arose due to their excellent wetting and fine-scale microstructural uniformity.

Cycle stability was assessed at 2 C over 400 cycles, as shown in Figure 10b. All cells had an initial capacity of ∼155 mAh g^–1^ at 2 C on the first cycle (assumed as 100% capacity). After 400 cycles, Al_2_O_3_ (5 μm), Al_2_O_3_ (22 μm), and Celgard retained capacities of 97.5 ± 0.9, 96.0 ± 0.4, and 91.5 ± 1.4%, with a capacity loss per cycle of 0.0068, 0.0093, and 0.0210%, respectively. The 2–3 times greater capacity retention for cells using Al_2_O_3_ separators was again ascribed to their significantly lower impedance and improved wetting.

It is worth recalling that the sprayed electrodes in the half-cells tested here were not calendared. Nonetheless, even the 5 μm thick Al_2_O_3_ separator provided consistent, high performance enabled by the excellent conformal coating and even coverage.

### 3.4 Physical and Electrochemical Properties of Full Cells

Previous work has shown two advantageous properties of spray deposition: (1) the ability to process multiple layers in a continuous operation, due to “on-the-fly” changing of deposition conditions and incremental drying of layers,^42,43,68,45^ and (2) conformal coating of presprayed substrates. Combining these attributes could enable coating of LIBs onto a variety of substrates, including nonflat geometries.

**Figure 11:**
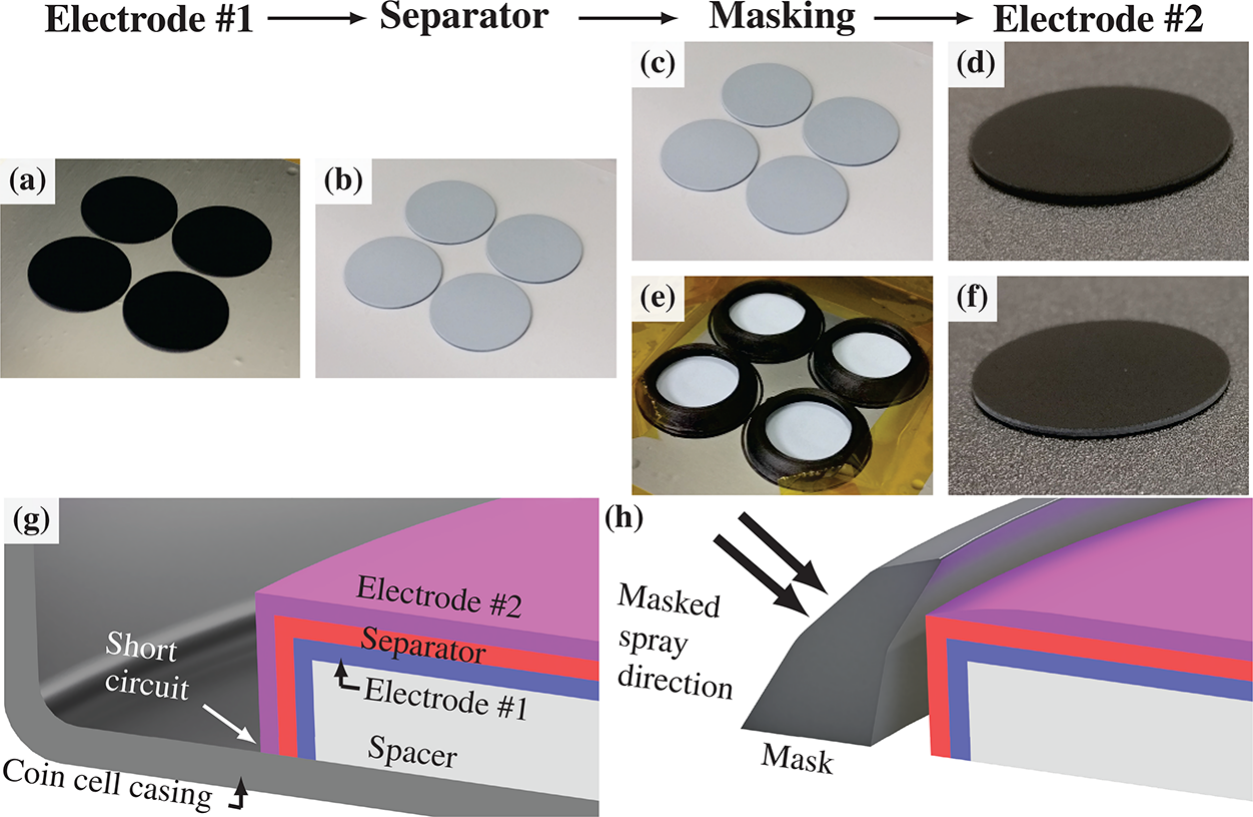
Process of manufacturing sequentially spray-deposited full cells: (a) initial electrode layer deposition onto spacers, (b) Al_2_O_3_ separator layer deposition, (c) second electrode deposition without mask, (d) final full cell with a conformal second electrode, (e) second electrode deposition with 3D printed masks, and (f) final full cell with second electrode restricted from conformal coating. Schematic cross sections of (g) the full cell without second electrode layer masking and (h) the full cell with second electrode layer masking and the masking process.

Figure 11 shows the processing steps for manufacturing Li-ion full cells by spray deposition: (a) electrode #1 (LTO-based) was deposited onto spacers, (b) an Al_2_O_3_ separator was deposited onto electrode #1, (e) 3D printed masks were positioned over the LTO/Al_2_O_3_-coated spacers, and (f) electrode #2 was deposited onto masked LTO/Al_2_O_3_. Figure 11c,d shows how deposition without masks resulted in conformal coating of electrode #2 onto the side of the LTO/Al_2_O_3_-coated spacers, leading to short circuits on cell assembly, as shown in the schematic diagram in Figure 11g. Figure 11f shows the separator layer on the side of the spacer visible even after deposition of electrode #2, which prevented shorting because of the masking arrangement shown schematically in Figure 11h.

**Figure 12:**
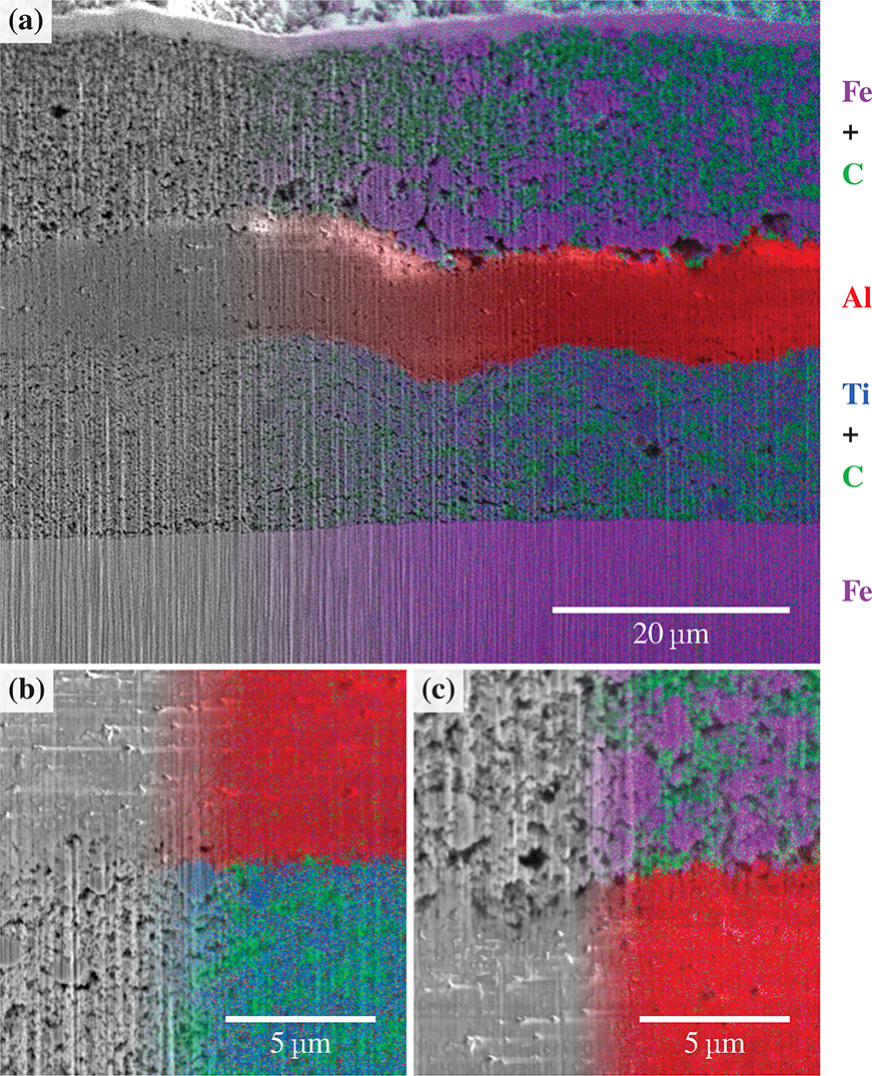
Cross-sectional SEM image of a spacer/LTO/Al_2_O_3_ separator/LFP cell assembly with an overlaid EDX map on the right-hand side of each image. The layers measure approximately 14.8 ± 2, 9.7 ± 0.7, and 17.7 ± 1.7 μm in thickness for LTO, Al_2_O_3_ separator, and LFP, respectively. The EDX element color key is displayed on the right of the image.

Cross sections of an Al_2_O_3_ separator full cell were examined by SEM imaging and EDX analysis, as shown in Figure 12. Ti (blue) differentiated the LTO, Al (red) the Al_2_O_3_ separator, Fe (purple) the LFP and stainless steel spacer, and C (green) in both the LTO (anode) and LFP (cathode). In this case the LTO-based layer had slightly larger variations in thickness of up to 4 μm. Nonetheless, the 10 μm Al_2_O_3_ separator layer conformally adopted the surface topography. The LTO/separator interface had excellent contact while the separator/LFP interface had some pores of 2–3 μm diameter.

Figure 12b,c displays magnified cross sections of (b) the LTO/separator interface and (c) the separator/LFP interface. Overall, all the layers showed remarkably good particle packing and consistency of microstructure given the absence of calendering.

**Figure 13:**
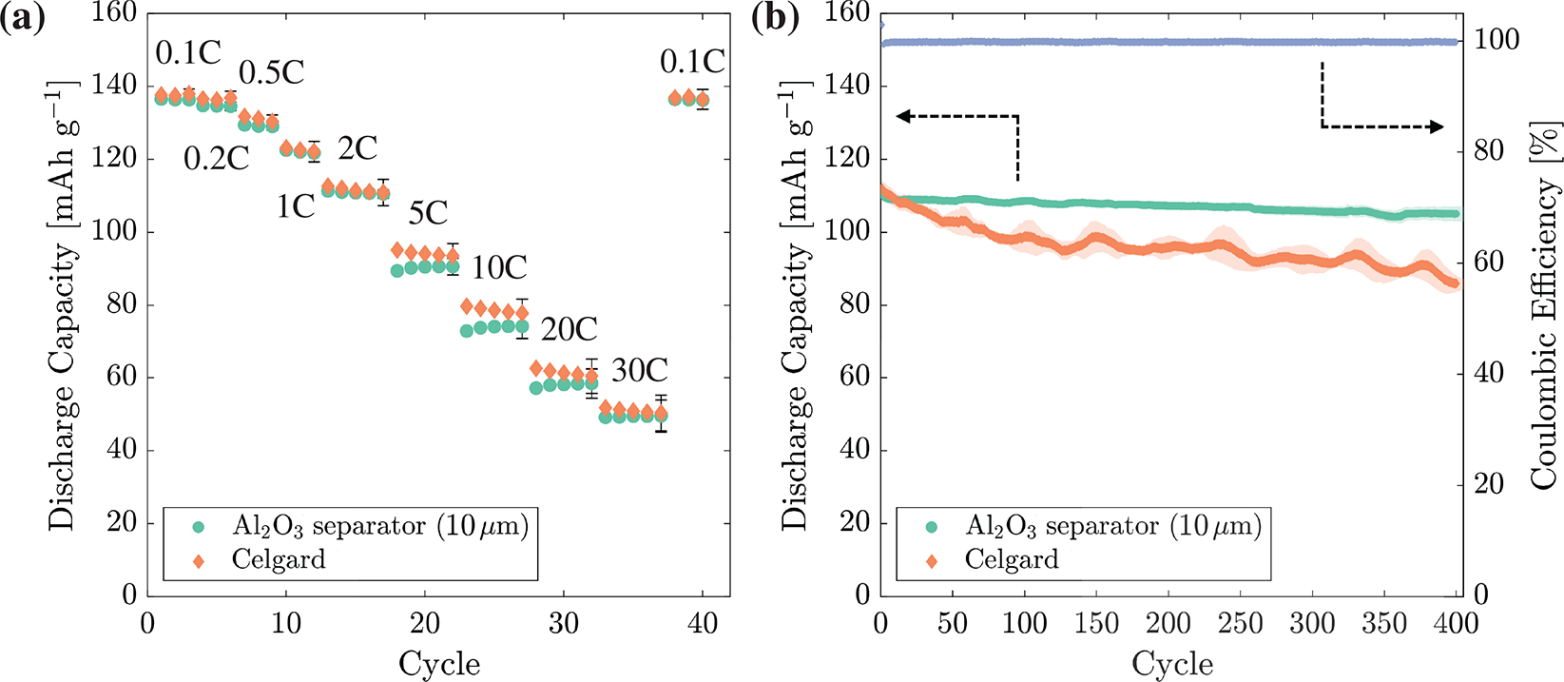
Electrochemical rate and cycling testing of spacer/LTO/separator/LFP full cells displaying discharge capacity of the LFP electrode. (a) Rate testing of full cells manufactured by using continuous spray deposition with an Al_2_O_3_ separator or conventional assembly using a polypropylene Celgard 2500 separator between 0.1 and 30 C. (b) Cycle stability of cells over 400 cycles at 2 C.

Figure 13a shows full cell rate testing performed between 0.1 and 30 C, with discharge capacity determined in terms of the LFP cathode for both the Al_2_O_3_ separator full cells and conventional full cells using a polypropylene Celgard 2500 separator. The cells were fabricated with an LFP:LTO capacity ratio of 1:1 according to 0.1 C capacities measured previously, and all electrodes were manufactured by using spray deposition. The Celgard full cells had a capacity of 137.2 ± 0.4 mAh g^–1^ at 0.1 C, while those with a Al_2_O_3_ separator had a similar capacity of 136.3 ± 0.8 mAh g^–1^. Between 5 and 30 C, the Celgard 2500 separator enabled a greater capacity at the start of each set of five cycles, but capacity reduced each cycle. On the other hand, the cells using a Al_2_O_3_ separator increased in capacity during each set of cycles until capacities were almost equal. After 30 C, Celgard and Al_2_O_3_ separator full cells recovered to capacities of 134.8 ± 0.5 and 136.2 ± 0.8 mAh g^–1^ at 0.1 C, respectively.

Cycle stability was examined over 400 cycles at 2 C directly after the rate testing. Celgard and Al_2_O_3_ separator full cells had initial capacities of 108.6 ± 1.5 and 109.0 ± 1.3 mAh g^–1^, reducing to 86.0 ± 1.4 and 104.9 ± 1.9 mAh g^–1^ after 400 cycles. The average Coulombic efficiency of each cell was 99.8%. After 400 cycles, Celgard and Al_2_O_3_ separator full cells had final capacities of 79.2 ± 1.3 and 96.3 ± 1.8% and a capacity loss per cycle of 0.0354 and 0.0106%, respectively.

Relating the degradation of Celgard full cell capacity to that of LTO/Celgard half-cells (Figure 10), the capacity loss per cycle calculated by linear regression for Celgard full cells was almost 1.7× greater over 400 cycles. The capacity loss per cycle of full cells with a 10 μm thick Al_2_O_3_ separator after 400 cycles, 0.0106%, was slightly larger than 5 and 22 μm thick Al_2_O_3_ separator half-cells with 0.0068 and 0.0093%, respectively. However, the capacity of the LFP was intrinsically less stable than the LTO under long-term cycling, so a poorer capacity retention in the LFP/LTO full cells was expected.

Overall, the best sprayed Al_2_O_3_-based separators performed at least as well as the commercial Celgard polymer separator, but with notable improvements in cell capacity retention in integrated full cells and high C-rate response in LTO half-cells. A significant reduction in separator thickness was evidenced for the integrated full cell compared to previous literature (from 200 to 5−10 μm).^41^ Several optimizations have been suggested to contribute to the effectiveness of the sprayed Al_2_O_3_ separators. The mixed carrier liquid improved uniformity of deposited particles by influencing droplet evaporation, the small average separator pore size (50 nm) blocked penetration of conducting material from the second electrode layer or Li metal, and the combination of high substrate temperature and the addition of SBR emulsion stabilized layers of separator material upon deposition. The Al_2_O_3_ separator in the full cell assembly was thinner than the Celgard separator, which contributed to the difference in capacity retention partly. However, as shown in Figure 10b, the Al_2_O_3_ separator with similar thickness to the Celgard separator displayed significantly better capacity retention in a half-cell configuration. Therefore, it is suggested that the principal reason for the slower degradation of the Al_2_O_3_ separator containing cells is their intrinsic improved wetting of the electrolyte (Figure 8) and lower impedance (Figure 9). The lower impedance reduces the required overpotentials to drive electrochemical energy storage reactions, and so degradation by unwanted side-reactions that may occur at elevated potentials and temperatures is somewhat reduced.

The spray deposition route can also be used to fabricate successfully LIB anodes and cathodes from a variety of different active materials, including those with particles diameters of up to 20 μm. When using larger diameter particles for spray-deposited three-layer full cells in a single operation, i.e., similar to the LFP/Al_2_O_3_/LTO cells fabricated here, the unavoidable increase in surface roughness associated with larger diameters can cause difficulties in ensuring the subsequent sprayed separator layer provides an unbroken electron barrier of approximately constant thickness over the entire electrode area. The most effective approach to this challenge is to introduce an intermediate calendaring step that effectively smooths the sprayed surface (as well as providing some electrode densification). Although this undermines some of the single operation benefits of the current arrangements, it is relatively simple and effective to implement. For convenience, the spray deposition process is normally operated with aqueous based suspensions/solvents, although it can also be successfully implemented in an inert, dry atmosphere by using organic solvents for more air/water sensitive active materials. Solid electrolytes have also been successfully codeposited for hybrid solid state cathodes and separators based on ion-conducting polymers and inorganic electrolytes.^44,69^ The ability to control surface topography to mitigate separator defects is also useful as larger area electrodes and one-step cells are fabricated in scale-up investigations. In this ongoing work, calendering is also used to add the top-layer or “upper” current collector with sufficient cycle life stability and low impedance, for example, by warm rolling of an Al foil precoated with a thin layer of thermopolymer onto the three-layer assembly.^70^ However, it should be noted that calendering of multilayers (as opposed to calendering layer by layer) may develop a tendency for short circuits to form across the separator should there be regions of unusually high surface roughness.

## 4 Conclusions

Spray-deposited Al_2_O_3_-based separators enabled stable, high rate, and long-term cycling of LTO/Li and LFP/LTO cells. Initial optimization of dispersant, binder, and solvent for spray-deposited separators assessed suspension stability and deposition properties. An optimized Al_2_O_3_-based separator contained 1 wt % PAA and 5 wt % SBR and was deposited from an aqueous suspension containing 20 vol % IPA. Al_2_O_3_ separator surface morphology was more uniform with a 20 vol % IPA solvent than with water alone, attributed to a greater Marangoni flow during droplet drying that more efficiently distributed the 50 nm particles into a relatively uniform layer.

Al_2_O_3_ separators between 5 and 22 μm thick had consistent and similar porosity of ∼58%, excellent wettability (contact angle ∼0°), thermal stability to at least 180 °C, adequate electrochemical stability, and high ionic conductivity of 0.98 mS cm^–1^, double that of a commercial PP separator. Furthermore, LTO half-cells with 5 and 22 μm Al_2_O_3_ separators showed improved rate performance and excellent cycle stability (0.007–0.009% capacity fade per cycle) compared with Celgard 2500 (0.021% capacity fade per cycle) due to the improved compatibility of the electrolyte and separator.

A full cell manufactured in a sequential spray process with a 10 μm Al_2_O_3_ separator, the first of its kind, showed similar rate performance to a conventional Celgard 2500 full cell with a capacity of ∼50 mAh g^–1^ at 30 C. Upon long-term cycling at 2 C, the Al_2_O_3_ separator full cells displayed excellent capacity retention per cycle of 0.0106% over 400 cycles compared with 0.0354% for the full cells with a Celgard separator. After 400 cycles, Al_2_O_3_ and Celgard separator full cells retained ∼96 and ∼79% of their initial 2 C capacity due to the lower impedance of the Al_2_O_3_ separator in the full cell assembly.

Further avenues to improve the oxide separator performance include selecting particles with lower density, improving the separator drying step, and using a dispersant with better dispersive and thermal properties than PAA. Nonetheless, this work showed that a single deposition technique can be used to process both electrodes and separators for Li-ion batteries and that cells manufactured by using this assembly technique can have similar or greater performance than those assembled with a conventional PP separator.
